# Warm-up stretching exercises and physical performance of youth soccer players

**DOI:** 10.3389/fphys.2023.1127669

**Published:** 2023-02-15

**Authors:** Jordan Hernandez-Martinez, Rodrigo Ramirez-Campillo, Tiago Vera-Assaoka, María Castillo-Cerda, Bastian Carter-Thuillier, Tomás Herrera-Valenzuela, Antonio López-Fuenzalida, Hadi Nobari, Pablo Valdés-Badilla

**Affiliations:** ^1^ Universidad de Los Lagos, Osorno, Chile; ^2^ Department of Physical Activity Sciences, Universidad de Los Lagos, Osorno, Chile; ^3^ Programa de Investigación en Deporte, Sociedad y Buen Vivir, Universidad de Los Lagos, Osorno, Chile; ^4^ Exercise and Rehabilitation Sciences Laboratory, School of Physiotherapy, Faculty of Rehabilitation Sciences, Universidad Andres Bello, Santiago, Chile; ^5^ Department of Education, Universidad de Los Lagos, Osorno, Chile; ^6^ Universidad Católica de Temuco, Temuco, Chile; ^7^ School of Physical Activity, Sports and Health Sciences, Faculty of Medical Sciences, Universidad de Santiago (USACH), Santiago, Chile; ^8^ Department of Rehabilitation, Intervention and Therapeutic Approach, School of Health Sciences, Universidad de Playa Ancha, Valparaíso, Chile; ^9^ Universidad Andrés Bello, Viña del Mar, Chile; ^10^ Faculty of Sport Sciences, University of Extremadura, Cáceres, Spain; ^11^ Department of Motor Performance, Faculty of Physical Education and Mountain Sports, Transilvania University of Brașov, Brașov, Romania; ^12^ Department of Physical Activity Sciences, Faculty of Education Sciences, Universidad Católica del Maule, Talca, Chile; ^13^ Carrera de Entrenador Deportivo, Escuela de Educación, Universidad Viña del Mar, Viña del Mar, Chile

**Keywords:** plyometric exercise, muscle strength, team sport, muscle stretching exercises, adolescent development

## Abstract

This study aims to compare the effects of standard warm-up versus warm-up using stretching exercises on the physical performance of male youth soccer players. Eighty-five male soccer players (age: 10.3 ± 4.3 years; body mass index: 19.8 ± 4.3 kg/m^2^) were assessed for countermovement jump height (CMJ, cm), 10 m, 20 m and 30 m running sprint speed (s) and ball kicking speed (km/h) for the dominant and non-dominant leg under five (randomized) warm-up conditions. Using 72 h of recovery between conditions, the participants completed a control condition (CC) and four experimental conditions, including static stretching (SSC), dynamic stretching (DSC), ballistic stretching (BSC), and proprioceptive neuromuscular facilitation (PNFC) exercises. All warm-up conditions had a duration of 10 minutes. The main results indicate that no significant differences (*p* > 0.05) were found between warm-up conditions compared to CC in CMJ (CC = 28.1 ± 4.9; SSC = 28.4 ± 4.9; DSC = 30.9 ± 4.8; BSC = 30.9 ± 5.2; PNFC = 28.4 ± 5.0), 10 m sprint (CC = 2.42 ± 0.4; SSC = 2.50 ± 0.4; DSC = 2.30 ± 0.3; BSC = 2.27 ± 0.3; PNFC = 2.53 ± 0.4), 20 m sprint (CC = 5.42 ± 0.9; SSC = 5.59 ± 0.9; DSC = 5.37 ± 0.9; BSC = 5.40 ± 0.9; PNFC = 5.44 ± 0.9), 30 m sprint (CC = 8.05 ± 1.3; SSC = 8.27 ± 1.3; DSC = 8.01 ± 1.3; BSC = 8.00 ± 1.3; PNFC = 8.12 ± 1.3), ball kicking speed for dominant (CC = 56.2 ± 4.9; SSC = 55.3 ± 5.2; DSC = 56.9 ± 5.8; BSC = 57.3 ± 5.8; PNFC = 55.7 ± 5.2) and non-dominant leg (CC = 52.8 ± 3.4; SSC = 51.8 ± 4.6; DSC = 53.5 ± 5.4; BSC = 53.6 ± 4.9; PNFC = 52.5 ± 4.0). In conclusion, compared to standard warm-up, stretching-based warm-up exerts no effect on male youth soccer players jump height, sprint speed and ball kicking speed.

## Introduction

Soccer is a sport that requires intermittent high-intensity actions (approximately 600 actions and about 40 high-intensity actions >21 km/h), such as running, jumping and ball striking (Dolci et al., 2020), being determinants for success in this sport (Low et al., 2020). In official matches, male soccer players perform between 150 and 250 intense actions interspersed with periods of low-intensity actions (Low et al., 2020). The actions aim to end with scoring a goal whose average is 2.66. In European leagues, teams that score the first goal have a higher percentage (65%–70%) probability of winning the game (Martínez and García, 2019). For example, most goals are preceded by linear speed (45%) and vertical jumps (16%) (Faude et al., 2012; Emmonds et al., 2019; González-Rodenas et al., 2020). The aforementioned actions are associated with high levels of strength, power and sprint speed, determinant factors for successful participation in youth and adult soccer players (Emmonds et al., 2019; González-Rodenas et al., 2020). Considering the importance of the explosive actions performed during the matches (Low et al., 2020), it is essential to execute actions that lead to maximize physical performance in soccer players such as warm-up (Hammami et al., 2018). Scientific evidence has reported the effectiveness of different types of warm-up on physical-specific conditions (van den Tillaar et al., 2019; van den Tillaar and von Heimburg, 2016; Chaâri and Frikha, 2022), which necessarily implies defining correctly the pre-match warm-up according to the characteristics of the soccer players (e.g., age, level, experience, hours of training). These warm-up sessions include short-duration, high-intensity activities that aim to improve physical fitness sat higher efforts by increasing intramuscular temperature, nerve conductance rate, and metabolic reactions (Hammami et al., 2018). It is well documented that muscle performance can improve by 3.46%–4.21% acutely after specific warm-up in muscle strength, speed and jumping by 1%–20% in adult soccer players (Hammami et al., 2018; Isla et al., 2021a).

Including stretching exercises in the warm-up (static, dynamic or ballistic) reduces the incidence of injury, accelerates recovery and improves physique-specific performance (Hammami et al., 2018; Gil et al., 2019). In a systematic review by Silva et al. (2018), it was observed that warm-up by dynamic stretching leads to improvements in sprints of 7.6%, 6.6% in agility and, 8.6% in vertical jump height in team sports. While in professional soccer players Vazini Taher and Parnow (2017) observed that static stretching led to a 2.8% decrease in jump height through countermovement jump (CMJ) compared to dynamic stretching in professional soccer players. Similarly, Vasileiou et al. (2013) observed that dynamic stretching led to a statistically significant (*p* < 0.05) improvement in 20 m maximal speed performance by 0.02%–0.06% decrease by static stretching in amateur soccer players. Similar results were presented by Gelen (2010), who showed that dynamic stretching led to a statistically significant (*p* < 0.05) increase in ball striking speed of 3.3%, while static stretching presented a decrease of 2.1% in professional soccer players. In contrast, Oliveira et al. (2018) reported decreases in CMJ by warm-ups with static stretching (2.3%), proprioceptive neuromuscular facilitation (2.8%), active stretching (0.4%) and ballistic stretching (0.7%) along with decreases in 10 m (0.01%, 0.04%, 0.01% and 0.02%) and 20 m (0.01%, 0.06%, 0.04% and 0.02%) running sprint speed in youth soccer players when compared to a control group that performed a traditional warm-up (without stretching exercises). Including stretching static, dynamic, ballistic and proprioceptive neuromuscular facilitation has decreased the risk of injury, increased range of motion and greater neuromuscular activation (Behm et al., 2016; Oliveira et al., 2018).

Considering the importance of performing an optimal warm-up to improve performance on variables such as jump height, sprint speed, and ball kicking speed in soccer players, along with the discrepancies in the literature and the paucity of scientific literature addressing the implications of stretching during warm-up in youth soccer players, the present study aims to compare the effects of standard warm-up versus warm-up with stretching exercises (e.g., static, dynamic, ballistic, and proprioceptive neuromuscular facilitation) on jump height, sprint speed, and ball kicking speed in male youth soccer players. Base on previous studies (Behm et al., 2016; Oliveira et al., 2018) we hypothesized that warm-up by stretching has no significant effect on jump height, sprint speed and ball kicking speed in youth soccer players.

## Materials and methods

### Study design and participants

**FIGURE 1:**
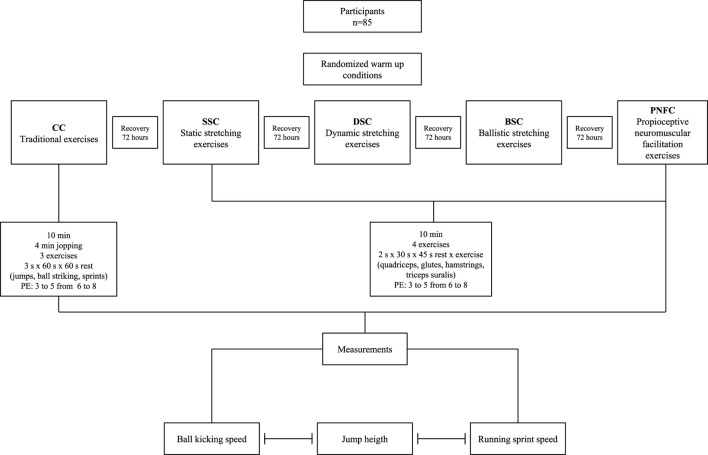
Study design. CC: control condition, traditional exercises (movements in different directions with progressive increases in speed). SSC: static stretching condition, static stretching exercises (static stretching movements with progressive increases in amplitude). DSC: dynamic stretching condition, dynamic stretching exercises (oscillatory stretching movements with progressive increases in speed). BSC: ballistic stretching condition, ballistic stretching exercises (hold 5 s elongated position than 5 s of oscillatory movement with progressive increments completing 30 s). PNFC: proprioceptive neuromuscular facilitation condition, neuromuscular facilitation exercises (passive stretching 10 s followed by 10 s of isometric contraction followed by 10 s of passive stretching with progressive increases in intensity). PE: perception of effort.

Randomized crossover trial, with proportional sampling (https://www.randomizer.org) and homogeneous organized, in which a group of youth male soccer players participated in five warm-up conditions, one control (CC) (traditional warm-up, i.e., without flexibility exercises) and four experimental conditions using stretching exercises [static (SSC), dynamic (DSC), ballistic (BSC) and proprioceptive neuromuscular facilitation (PNFC)]. Participants performed all the randomized warm-up conditions with a rest between conditions of 72 h. After each warm-up condition, jump height, sprint speed, and ball kicking assessments were performed, summarized in Figure 1.

**FIGURE 2:**
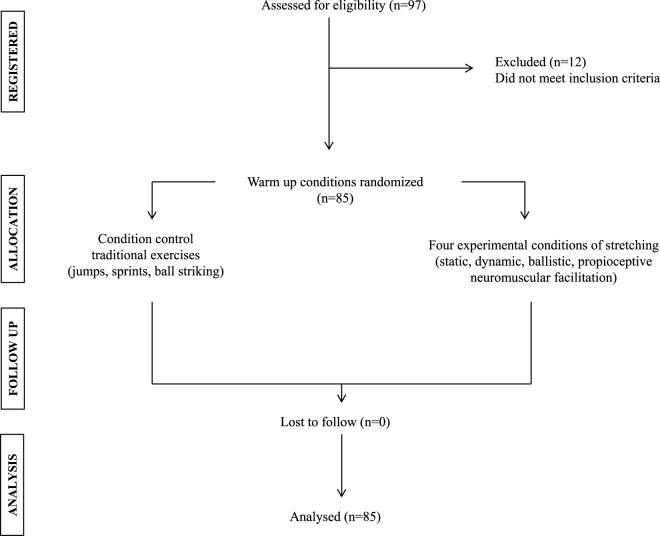
Flow chart of the recruitment process.

The sample size calculation indicates that the ideal number of participants per condition is 12, according to a previous study (Oliveira et al., 2018). An alpha level of 0.05 was considered with a power of 88% with an effect size of strong (*d* = 0.77). The GPower software (version 3.1.9.6, Franz Faul, Universiät Kiel, Germany) was used to calculate the statistical power. Eighty-five male soccer players (age: 13.3 ± 3.4 years, body mass: 40.1 ± 3.4 kg, bipedal height: 1.42 ± 2.8 m, body mass index (BMI): 19.8 ± 4.3 m/kg^2^) from four soccer schools belonging to professional clubs in Chile participated. The inclusion criteria were: i) to be free of injuries that would prevent them from performing the warm-up conditions and physical performance assessments; ii) to have the appropriate sports clothing to carry out the procedures; iii) not to be training in other soccer schools or teams attached to the existing one; iv) not to be in competitions on the same days as the warm-up conditions were carried out. The exclusion criteria will be considered: i) those who presented cardiovascular or musculoskeletal pathologies that prevented them from carrying out the flexibility sessions or physical assessments; ii) not participating in all the warm-up sessions. There were no injuries or discomfort while performing the warm-up sessions and the physical performance assessments. The recruitment process to obtain the final sample size is presented in Figure 2. All participants and their proxies or legal guardians had to accept the criteria for the use and handling of the data by signing an assent and informed consent, respectively, authorizing the use of the information for scientific purposes. The research protocol was reviewed and approved by the Bioethics Committee of the University of Playa Ancha, Chile (approval number: 005/2016) and was developed following the guidelines of the Helsinki declaration 2013 regarding research involving human subjects.

### Assessments

#### Anthropometric measurements

Bipedal height was measured using the Frankfort plane in a horizontal position, with a tape measure (Bodymeter 206, SECA, Germany; accuracy to 0.1 cm) attached to the wall. Body mass was measured using an electronic scale (Omron HBF 514: accuracy to 0.1 kg Osaka, Prefectura de Osaka, Japón), while BMI was calculated by dividing body mass by bipedal height squared (kg/m^2^). All measurements were performed following the recommendations of the International Society for Advances in Kinanthropometry (ISAK) (Marfell-Jones et al., 2012).

#### Jump height

It was measured through the CMJ according to previous recommendations (Bosco et al., 1983). For the CMJ, soccer players executed maximal effort jumps on a mobile contact platform Ergojump® Globus, (ErgoTest, Codogne, Italy) with arms on the iliac crests. Take-off and landing were standardized at the exact location, and players executed entire knee and ankle extensions during the flight phase. The best of three jumps was recorded with a 1-min rest between each attempt. The test-retest was used to determine reliability. The data obtained for the vertical jump height by CMJ was determined at very high reliability of 0.99.

#### Running sprint speed

Sprint time was assessed to the nearest 0.01 s using single-beam timing gates Brower® Timing System, (Salt Lake City, Utah, United States). Participants started by placing behind the starting line the preferred toe-off. The sprint began when the player initiated the test, automatically triggering the timing. Timing gates were placed at the start (0.3 m in front of the athlete) and 10 m, 20 m, and 30 m. They were placed ∼0.7 m above the floor (approximately hip level). This system allows capturing trunk movement rather than a false trigger of a limb. Three sprints were performed, recording the best of the three with a 1-min rest between each attempt (Drozd et al., 2017). The data obtained for the maximum speed at 10 m, 20 m and 30 m was determined at high reliability of 0.98.

#### Ball kicking speed

Participants performed a maximal instep ball strike with their dominant and non-dominant legs after a two-stride run using a size five soccer ball (Adidas Starlancer V®, FIFA certified, Herzogenaurach, Baviera, Alemania). Maximum speed was measured with a radar gun Speed Gun SR3600 (Sports Radar®, Homosassa, Florida, United States). Three attempts were carried out, recording the best of the three with a 1-min rest between each attempt (Ramírez-Campillo et al., 2015). The data obtained for ball striking speed was determined to have high reliability of 0.94.

#### Intervention (warm-up conditions)

The CC had a traditional soccer warm-up for 10 min composed of jogging for 4 min in different directions with moderate-to vigorous-intensities measured with the ten-point rating of perceived exertion (RPE) (Borg, 1982) that started between 3 and 5 points and ended between 6 and 8 points, followed by movements executed in matches (jumps, ball striking, change of direction movements) distributed in three sets of 60 s each with a 60 s rest between sets. This condition did not consider flexibility exercises.

The SSC consisted of a warm-up using static stretching for 10 minutes. Four stretching exercises were performed, one for each muscle group of the lower body (quadriceps, gluteus, hamstrings and triceps suralis) distributed in two series of 30 s each with a rest of 45 s per exercise, executing progressive increases in the amplitude of joint movement, intensity started between 3 and 5 points, ending between 6 and 8 points of RPE.

The DSC consisted of a 10-min warm-up with dynamic stretching. Four stretching exercises were performed, one for each muscle group of the lower body (Quadriceps, Gluteus, Hamstrings and Triceps suralis) distributed in two series of 30 s each with a rest of 45 s per exercise, dynamically executing oscillatory stretching movements with progressive increases in speed. The intensity started between 3 and 5 points, ending between 6 and 8 points of RPE.

The BSC consisted of a warm-up with ballistic stretching for 10 min. Four stretching exercises were performed, one for each muscle group of the lower body (Quadriceps, Gluteus, Hamstrings and Triceps suralis) distributed in two series of 30 s each with a rest of 45 s per exercise in which an elongated position was maintained for 5 s then 5 s of oscillation with progressive increments until completing the 30 s of each series. The intensity started between 3 and 5 points, ending between 6 and 8 points of RPE.

The PNFC consisted of a warm-up through proprioceptive neuromuscular facilitation for 10 minutes. Four stretching exercises were performed, one for each muscle group of the lower body (Quadriceps, Gluteus, Hamstrings and Triceps suralis) distributed in series of 30 s each with a rest of 45 s per exercise using the retention-relaxation technique (stretching), which consists of 10 s of passive stretching followed by 10 s of isometric contraction of the stretched muscle, followed by 10 s of passive flexibility applied by a professional in the area of physical activity sciences with progressive increases in intensity starting between 3 and 5 points and ending between 6 and 8 points of RPE.

#### Statistical analyses

Values were reported as mean ± standard deviation. The Kolmogorov-Smirnov test was used to determine the normality of the data, while the Levene’s test was used to determine the homogeneity of variance. Normal distribution was observed for all data. To compare the physical performance variables based on the type of intervention used the one-way ANOVA test with Bonferroni’s *post hoc*. The effect size (ES) was calculated with Cohen’s d (Cohen, 1992) considering a small (0.20–0.49), moderate (0.50–0.79) or strong (>0.80) effect the formula used was *d*= (M_1_-M_2_)/SD (Rendón-Macías et al., 2021). In all cases, a significance value of *p* < 0.05 was established. The STATISTICA 8 program was used to perform the statistical analysis.

## Results

No adverse effects or injuries were observed. No statistically significant differences (*F* = 0.86; *p* = 0.64) were observed in the jump height (CMJ) between heating conditions. However, there was a moderate ES in favor of DSC (*d* = 0.57), BSC (*d* = 0.55) and an insignificant ES in SSC and PNFC (*d* = 0.06) concerning CC, in addition to a magnitude of change located between 1% and 9.9%.

Running sprint speed reported no statistically significant differences for the 10 m (*F* = 2.35; *p* = 0.053), 20 m (*F* = 0.24; *p* = 0.90) and 30 m (*F* = 0.15; *p* = 0.95) sprint between warm-up conditions. However, a moderate ES in favor of BSC (*d* = 0.56) and a small ES in favor of SSC (*d* = 0.25), DSC (*d* = 0.28) and PNFC (*d* = 0.25) were present concerning the CC for the 10 m sprint, while the magnitude of change was between 4.1% and 8.3%. In the 20 m sprint, there was a small ES in favor of SSC (*d* = 0.25), an insignificant ES in DSC (*d* = 0.11) and no differences were reported in BSC and PNFC (*d* = 0.00) to CC, while the magnitude of change was between 1.8% and 3.7%. In the 30 m sprint, an insignificant ES was presented in SSC (*d* = 0.15), PNFC (*d* = 0.07) and no differences were reported in DSC and BSC (*d* = 0.00) to CC, while the magnitude of change was located between 1.2% and 2.5%.

Ball kicking speed did not present statistically significant differences for dominant (*F* = 0.56; *p* = 0.69) and non-dominant foot (*F* = 0.66; *p* = 0.62). Ball kicking speed with the dominant foot reported a small ES in favor of BSC (*d* = 0.20) and an insignificant ES in SSC (*d* = 0.17), DSC (*d* = 0.13) and PNFC (*d* = 0.09) with respect to CC, with a magnitude of change located between 0.8% and 1.9%. Ball kicking speed with the non-dominant foot presented a small ES in favor of SSC (*d* = 0.24) and an insignificant ES in BSC (*d* = 0.18), DSC (*d* = 0.15) and PNFC (*d* = 0.08) with respect to CC, with a magnitude of change located between 0.5% and 1.8%. The results of physical performance according to the warm-up conditions are presented in Table 1, while the differences between conditions are presented in Table 2.

**TABLE 1:** Physical-specific performance of youth’s male soccer players according to warm-up conditions.

Soccer players (*n* = 85)	CC (*n* = 85	SSC (*n* = 85)	DSC (*n* = 85)	BSC (*n* = 85)	PNFC (*n* = 85)	*F* value	*p*-value
CMJ (cm)	28.1 ± 4.9	28.4 ± 4.9	30.9 ± 4.8	30.9 ± 5.2	28.4 ± 5.0	0.86	0.64
Speed 10 (s)	2.43 ± 0.4	2.50 ± 0.4	2.30 ± 0.3	2.27 ± 0.3	2.53 ± 0.4	2.35	0.053
Speed 20 (s)	5.42 ± 0.9	5.59 ± 0.9	5.37 ± 0.9	5.40 ± 0.9	5.44 ± 0.9	0.24	0.90
Speed 30 (s)	8.05 ± 1.3	8.27 ± 1.3	8.01 ± 1.3	8.00 ± 1.3	8.12 ± 1.3	0.15	0.95
Ball kicking speed of the dominant foot (km/h)	56.2 ± 4.9	55.3 ± 5.2	56.9 ± 5.8	57.3 ± 5.8	55.7 ± 5.2	0.56	0.69
Ball kicking speed of the non-dominant foot (km/h)	52.8 ± 3.4	51.8 ± 4.6	53.5 ± 5.4	53.6 ± 4.9	52.5 ± 4.0	0.66	0.62

CC, control condition; SSC, static stretching condition; DSC, dynamic stretching condition; BSC, ballistic stretching condition; PNFC, proprioceptive neuromuscular facilitation condition.

**TABLE 2:** Differences between intervention conditions of youth’s male soccer players.

Intervention conditions (*n* = 85)	CMJ (cm)	Speed 10 (s)	Speed 20 (s)	Speed 30 (s)	Ball kicking speed of the dominant foot (km/h)	Ball kicking speed of the non-dominant foot (km/h)
CC vs. SSC	*d* = 0.06^a^ *p* = 1.00 (1.06%)	*d* = 0.17^a^ *p* = 1.00 (2.88%)	*d* = 0.18^a^ *p* = 0.90 (3.13%)	*d* = 0.16^a^ *p* = 0.95 (2.73%)	*d* = 0.17^a^ *p* = 0.60 (1.60%)	*d* = 0.24^b^ *p* = 0.92 (1.89%)
CC vs. DSC	*d* = 0.57^c^ *p* = 0.49 (9.96%)	*d* = 0.36^b^ *p* = 0.62 (5.34%)	*d* = 0.05^a^ *p* = 0.90 (0.92%)	*d* = 0.03^a^ *p* = 0.95 (0.49%)	*d* = 0.13^a^ *p* = 0.60 (1.24%)	*d* = 0.15^a^ *p* = 0.95 (1.32%)
CC vs. BSC	*d* = 0.55^c^ *p* = 0.46 (9.96%)	*d* = 0.45^b^ *p* = 0.33 (6.58%)	*d* = 0.02^a^ *p* = 0.90 (0.36%)	*d* = 0.03^a^ *p* = 0.95 (0.62%)	*d* = 0.20^b^ *p* = 0.65 (1.95%)	*d* = 0.18^a^ *p* = 0.90 (1.51%)
CC vs. PNFC	*d* = 0.06^a^ *p* = 0.16 (1.06%)	*d* = 0.25^b^ *p* = 1.00 (4.11%)	*d* = 0.02^a^ *p* = 0.90 (0.36%)	*d* = 0.03^a^ *p* = 0.95 (0.62%)	*d* = 0.09^a^ *p* = 0.65 (0.88%)	*d* = 0.08^a^ *p* = 0.90 (0.56%)
SSC vs. DSC	*d = 0.51* ^c^ *p* = 0.85 (8.80%)	*d* = 0.56^c^ *p* = 0.95 (8%)	*d* = 0.24^b^ *p* = 0.90 (3.93%)	*d* = 0.2^a^ *p* = 1.00 (3.14%)	*d* = 0.29^b^ *p* = 0.65 (2.89%)	*d* = 0.33^b^ *p* = 0.60 (3.28%)
SSC vs. BSC	*d = 0.49* ^b^ *p* = 0.79 (8.80%)	*d* = 0.65^c^ *p* = 0.53 (9.2%)	*d* = 0.21^b^ *p* = 0.92 (3.39%)	*d* = 0.20^b^ *p* = 1.00 (3.26%)	*d* = 0.36^b^ *p* = 0.63 (3.61%)	*d* = 0.37^b^ *p* = 0.60 (3.47%)
SSC vs. PNFC	*d = 0.00* ^a^ *p* = 1.00 (0%)	*d* = 0.07^a^ *p* = 1.00 (1.2%)	*d* = 0.16^a^ *p* = 0.91 (2.68%)	*d* = 0.11^a^ *p* = 1.00 (1.81%)	*d* = 0.07^a^ *p* = 0.70 (0.72%)	*d* = 0.16^a^ *p* = 0.60 (1.35%)
DSC vs. BSC	*d = 0.00* ^a^ *p* = 1.00 (0%)	*d* = 0.1^a^ *p* = 1.00 (1.30%)	*d* = 0.14^a^ *p* = 0.90 (2.46%)	*d* = 0.00^a^ *p* = 0.90 (0.12%)	*d* = 0.06^a^ *p* = 0.70 (0.70%)	*d* = 0.19^a^ *p* = 1.00 (0.18%)
DSC vs. PNFC	*d = 0.51* ^c^ *p* = 0.79 (8.09%)	*d* = 0.65^c^ *p* = 0.29 (10%)	*d* = 0.18^a^ *p* = 0.90 (3.22%)	*d* = 0.08^a^ *p* = 0.90 (1.37%)	*d* = 0.21^b^ *p* = 0.75 (2.10%)	*d* = 0.21^b^ *p* = 1.00 (1.86%)
BSC vs. PNFC	*d* = 0.49^b^ *p* = 0.73 (8.09%)	*d* = 0.73^c^ *p* = 1.00 (11.4%)	*d* = 0.04^a^ *p* = 0.90 (0.74%)	*d* = 0.09^a^ *p* = 0.90 (1.47%)	*d* = 0.29^b^ *p* = 0.60 (2.79%)	*d* = 0.24^b^ *p* = 1.00 (2.05%)

CMJ: countermovement jump. CC: control condition. SSC: static stretching condition. DSC: dynamic stretching condition. BSC: ballistic stretching condition. PNFC: proprioceptive neuromuscular facilitation condition. *d*: effect size.

^a^ Insignificant effect (<0.20).

^b^ Small effect (0.20–0.49).

^c^ Moderate effect (0.50–0.79).

*p*: statistical significance. %: percentage of change.

## Discussion

The present study aimed to compare the effects of warm-up versus warm-up using stretching exercises on the physical performance of male youth soccer players. Among the main results, it was found that there are no statistically significant differences between the CC for the experimental conditions of stretching (static, dynamic, ballistic and proprioceptive neuromuscular facilitation). However, a moderate ES (*d* > 0.50) with a magnitude of change greater than 9.9% in CMJ, a small ES (*d* > 0.20) in 10 m, 20 m, and 30 m sprint with a magnitude of change of 4.1%, while ball kicking speed presented a small ES (*d* > 0.20) with a magnitude of change of 1.9% in favor of the experimental conditions, significantly, for SSC, BSC, PNFC compared to CC.

It is important to assess lower extremity power in soccer players as performance in vertical jumping ability which has been correlated with acceleration and speed in youth soccer players (Zahalka et al., 2021), these explosive actions during the matches in soccer, such as the ability to jump and run at high speed, are determinants of good performance (Myftiu and Dalip, 2021). Actions such as those indicated occur in more than 50% of goal situations (Sánchez et al., 2020). Therefore, it is essential to use warm-up strategies that favor the performance of soccer players for such actions during matches. In the study conducted by Vazini Taher and Parnow (2017) on youth soccer players, an improvement of 2.8% was detected by dynamic stretching compared to static stretching in CMJ. In the study by Oliveira et al. (2018) a trend of improvement in jump height measured through the squat jump in youth soccer players through warm-ups with stretching when compared to control condition was detected, being this improvement of 0.1% with ballistic stretching, 0.3% with dynamic stretching, 1.3% with proprioceptive neuromuscular facilitation and 2.3% with static stretching. In a review by Hammami et al. (2018) in youth and adult soccer players, it was observed that warm-up using ballistic and dynamic stretching led to an improvement in CMJ with a small ES (*d* = 0.31). It might be associated with neural or mechanical factors or a combination of both (Oliveira et al., 2018). Although no significant differences were reported in our study, a moderate ES was detected in DSC and BSC (*d* > 0.50) concerning CC with a 9.9% change in CMJ, an auspicious fact aligned with previously reported studies.

Straight-line sprinting is the most frequent action before goals, both for the player scoring and assisting (Chmura et al., 2022). In the present 10 m, 20 m and 30 m sprint was similar to the warm-up condition. In the study by Oliveira et al. (2018), improvements in 10 m speed were detected for the warm-up condition with static stretching, which showed improvements of 1% compared to the ballistic stretching condition and 3% compared to the proprioceptive neuromuscular facilitation condition. In the study by López Mariscal et al. (2021) in youth soccer players, improvements of 0.6% in 20 m speed and 2% in 30 m speed were identified by warm-ups with static stretching compared to the warm-up with ballistic stretching. Despite finding no significant differences between the warm-up conditions applied in our study, a small and moderate ES was detected in SSC, BSC and PNFC (*d* > 0.20) for the 10 m sprint, in addition to a small ES for SSC (*d* > 0.20) for the 20 m sprint for the CC, with a change located between 3.7% and 4.1%. The literature has reported on other groups of soccer players (Oliveira et al., 2018; López Mariscal et al., 2021).

To generate an increase in the probability of scoring a goal, the soccer player must reach the highest possible ball speed, which depends on several variables, such as speed of the foot at the moment of impact, as well as the quality of the ball kicking (Rađa et al., 2019). Being relevant a good kicking technique with both feet (dominant and non-dominant) and if the kick is faster it is less likely that the goalkeeper or opposing player has enough time to react (Rađa et al., 2019). Therefore, it is vital to choose the right way to warm-up in order to prepare the body of the soccer players so that they have a more remarkable ability to hit the ball without risk of injury. In addition, it is relevant to consider individual characteristics such as age, skill level, gender and experience of the soccer players to choose the exercises (Palucci Vieira et al., 2021). In a study conducted by Amiri-Khorasani and Ferdinands (2014) on youth soccer players, an increase in ball kicking speed of 5.1% was observed using warm-up with ballistic stretching and 6.3% with static stretching compared to the CC. Similar to what was observed by Gelen (2010) in youth soccer players with a 3.3% increase in ball kicking speed performance using ballistic stretching warm-up compared to static stretching warm-up. Our study did not report significant differences for or against the warm-up conditions with stretching. However, a small ES was detected in BSC and SSC (*d* > 0.20) for CC, with a change between 1.8% and 1.9% in ball-kicking speed for both feet, which would indicate greater performance after the warm-up with static and ballistic stretching. Increasing performance in ball kicking speed may increase the likelihood of success in soccer (Gelen, 2010; Amiri-Khorasani and Ferdinands, 2014).

Among the limitations of the study are: i) not including measurements of neurophysiological mechanisms to determine muscle activation to the various stimuli in the warm-up conditions; ii) not assessing the level of physical maturation of the participants; iii) not having females in the sample. Among the strengths of the study, we can mention the randomization of the warm-up conditions and the choice of exercises and stretching methods aligned with those most commonly used in soccer. Based on the results found in the present study, it can be indicated that the warm-up based on static, dynamic, ballistic or proprioceptive neuromuscular facilitation stretching does not affect jump height, running sprint speed and ball kicking speed in male youth soccer players. Future studies could deepen the characteristics of the most appropriate stretching exercises and the optimal warm-up time to promote physical performance at different levels and age ranges.

## Practical applications

Based on findings of the present study, when comparing the standard versus the stretching-based warm-up, no significant effects were detected on running sprint speed, jump height and ball kicking speed in male youth soccer players. In this sense, we recommend the use of warm-ups based on flexibility in youth soccer players during training, since the literature reinforces its inclusion to promote development, prevent injuries and, as was reported (Hammami et al., 2018; Gil et al., 2019), does not alter the specific performance in soccer players. Similarly, it is advisable to carry out actions that accommodate the players in order to maximize their performance in explosive actions (Fashioni et al., 2020) that predominate during the game and lead to greater recruitment and activation of type 2 fibers (Isla et al., 2021b) predominants in the soccer. On the other hand, a lower RPE in the warm-up is associated with less muscle fatigue, wich has a positive impact on physical performance (Yanci et al., 2019).

## Conclusion

When comparing the standard warm-up with the stretching-based warm-up (regardless of its modality, i.e. SSC, DSC, BSC, PNFC) it exerts no effect on the jump height, sprint running speed and ball kicking speed of male youth soccer players. The incorporation of flexibility exercises in the warm-up (and their modality) may depend on player preference.

## Data Availability

The raw data supporting the conclusion of this article will be made available by the authors, without undue reservation.
