# Corrigendum: Warm-up stretching exercises and physical performance of youth soccer players

**DOI:** 10.3389/fphys.2023.1191277

**Published:** 2023-03-31

**Authors:** Jordan Hernandez Martinez, Rodrigo Ramirez-Campillo, Tiago Vera-Assaoka, María Angélica Castillo Cerda, Bastian Carter-Thuillier, Tomás Herrera-Valenzuela, Antonio López-Fuenzalida, Hadi Nobari, Pablo Valdés-Badilla

**Affiliations:** ^1^ Universidad de Los Lagos, Osorno, Chile; ^2^ Department of Physical Activity Sciences, Universidad de Los Lagos, Osorno, Chile; ^3^ Programa de Investigación en Deporte, Sociedad y Buen Vivir, Universidad de Los Lagos, Osorno, Chile; ^4^ Exercise and Rehabilitation Sciences Laboratory, School of Physiotherapy, Faculty of Rehabilitation Sciences, Universidad Andres Bello, Santiago, Chile; ^5^ Department of Education, Universidad de Los Lagos, Osorno, Chile; ^6^ Universidad Católica de Temuco, Temuco, Chile; ^7^ School of Physical Activity, Sports and Health Sciences, Faculty of Medical Sciences, Universidad de Santiago (USACH), Santiago, Chile; ^8^ Department of Rehabilitation, Intervention and Therapeutic Approach, School of Health Sciences, Universidad de Playa Ancha, Valparaíso, Chile; ^9^ Universidad Andrés Bello, Viña del Mar, Chile; ^10^ Faculty of Sport Sciences, University of Extremadura, Cáceres, Spain; ^11^ Department of Motor Performance, Faculty of Physical Education and Mountain Sports, Transilvania University of Brașov, Brașov, Romania; ^12^ Department of Physical Activity Sciences, Faculty of Education Sciences, Universidad Católica del Maule, Talca, Chile; ^13^ Carrera de Entrenador Deportivo, Escuela de Educación, Universidad Viña del Mar, Viña del Mar, Chile

**Keywords:** plyometric exercise, muscle strength, team sport, muscle stretching exercises, adolescent development

In the published article, there was an error regarding the **affiliation(s)** for Jordan Hernandez-Martinez; Tiago Vera-Assaoka. As well as having **affiliation** 1 Universidad de Los Lagos, Osorno, Chile; 2 Department of Physical Activity Sciences, Universidad de Los Lagos, Osorno, Chile; they should also have “3 Programa de Investigación en Deporte, Sociedad y Buen Vivir, Universidad de Los Lagos, Osorno, Chile”.

In addition, there was an error regarding the **affiliation(s)** for Bastian Carter-Thuillier. As well as having **affiliation** 5 Department of Education, Universidad de Los Lagos, Osorno, he should also have “3 Programa de Investigación en Deporte, Sociedad y Buen Vivir, Universidad de Los Lagos, Osorno, Chile; 6 Universidad Católica de Temuco, Temuco, Chile”.

In the published article, an **author** name was incorrectly written as Bastian Carter-Trullier. The correct spelling is “Bastian Carter-Thuillier”.

The authors apologize for this error and state that this does not change the scientific conclusions of the article in any way. The original article has been updated.

